# Long-term outcomes in patients with endometrial cancer after sentinel lymph node biopsy versus lymphadenectomy alone: a meta-analysis

**DOI:** 10.3389/fonc.2025.1584447

**Published:** 2025-05-20

**Authors:** Sai Zhang, Juan Zheng, Dandan Zhu

**Affiliations:** Department of Obstetrics and Gynecology, General Hospital of Xuzhou Mining Group, Xuzhou, Jiangsu, China

**Keywords:** endometrial cancer, sentinel lymph node biopsy, lymphadenectomy, oncological outcome, overall survival, progression free survival, disease specific survival

## Abstract

**Objective:**

This study aimed to assess the prognosis of endometrial cancer (EC) patients after sentinel lymph node biopsy (SLNB) or lymph node dissection (LND) alone.

**Methods:**

EMBASE, PUBMED, COCHRANE, and WEB of SCIENCE were thoroughly searched for relevant articles until October 2024. The outcomes of interest encompassed overall survival (OS), progression-free survival (PFS), and disease-specific survival (DSS). Data analysis was made in STATA 18.0. The Newcastle-Ottawa Scale tool was leveraged to appraise study quality.

**Results:**

13 studies were included, involving 36621 EC patients. No difference was revealed in OS between SLNB and LND (HR=1.04, 95%CI: 0.80–1.33; P=0.789). In subgroup analyses, the SLNB group from survival curves had worse OS (HR=1.63, 95%CI: 1.04-2.56; P=0.035); the SLNB group with intermediate- to high-risk EC had better OS (HR=0.20, 95%CI: 0.08-0.49; P<0.001). No difference was revealed in PFS between SLNB and LND (HR=0.99, 95%CI: 0.76–1.28; P=0.927). SLNB had better PFS in Asia (HR=0.44, 95% CI: 0.20-0.98, P=0.046) and stage I-III EC (HR=0.46, 95% CI: 0.24-0.89; P=0.021). No statistical difference was found in DSS (HR=3.18, 95%CI: 0.91-11.07; P=0.069).

**Conclusion:**

SLNB is an effective alternative to conventional LND in either low- or intermediate-high-risk EC patients. However, due to the retrospective nature of most included studies and the limited data on high-risk patients, further prospective randomized controlled trials are warranted to validate these findings.

**Systematic review registration:**

https://www.crd.york.ac.uk/PROSPERO, identifier CRD42024489323.

## 1 Introduction

Endometrial cancer (EC) is the sixth most prevalent cancer in women worldwide (1). GLOBOCAN 2020 reported 417,367 new cases of EC and 97,370 deaths globally in 2021, and the incidence and mortality are increasing year by year (2). Surgery is currently the main treatment method for EC. According to the 2009 FIGO staging system for endometrial cancer, the survival rates of stage IA, stage IB, stage IIIC1, and stage IIIC2 EC are 89.6%, 77.6%, 57%, and 49%, respectively (3). Multiple factors affect EC prognosis, including histological type, pathological grading, myometrial infiltration, and lymph node metastasis (LNM) (4, 5). Among them, LNM affects the surgical pathological stage and may lead to recurrence and distant metastases, thereby reducing long-term survival (6). Hence, the accurate identification of LNM status intraoperatively is the key to EC surgery (7).

Traditional comprehensive surgical staging usually includes systematic pelvic and/or para-abdominal aortic lymph node dissection (LND) to determine the presence of LNM and to provide evidence to subsequent adjuvant therapy (8, 9). Nonetheless, although this extensive LND is helpful for accurate staging, it also poses a higher risk of complications, like lymphedema, infection, and nerve damage, which greatly affects the quality of life (10, 11). With the development of minimally invasive techniques and imaging in recent year, sentinel lymph node biopsy (SLNB) has gained attention as a more conservative surgical approach (12). SLNB is designed to identify and detect the lymph nodes that are the first to receive tumor drainage (i.e., the sentinel lymph nodes), thereby ensuring diagnostic accuracy while decreasing unnecessary LND and its associated side effects (13, 14). Some guidelines recommend SLNB for low-risk EC individuals, while systematic pelvic and aortic LND is still recommended for high-risk patients (15). Meanwhile, recent studies have stated that SLNB yields similar oncologic outcomes to LND (16, 17). Other studies have demonstrated significant differences in overall survival (OS) between SLNB and LND (18, 19), possibly related to the risk status in EC patients. Given the controversial findings in recent years, this meta-analysis aims to pool previous relevant studies to analyze the prognostic difference between SLNB and LND in EC patients, especially for high-risk EC patients.

## 2 Materials and methods

This paper was registered in the PROSPERO (CRD42024489323) and followed the PRISMA guidelines (20).

### 2.1 Search strategy

EMBASE, PUBMED, COCHRANE, and WEB of SCIENCE were thoroughly searched for relevant articles until October 2024. The search keywords encompassed “endometrial cancer”, “sentinel node”, and “lymphadenectomy”. The retrieved documents included reviews, meta-analyses, and some gray literature to ensure the comprehensive search. Details of search strategies are listed in **Supplementary Table S1**.

### 2.2 Eligibility criteria

Two reviewers (ZS and ZDD) selected eligible studies independently. Articles were eligible if they fulfilled the criteria: 1) EC individuals diagnosed by pathology; 2) comparing SLNB (whether with additional LND or not) and LND; 3) reporting OS and progression-free survival (PFS); 4) retrospective or prospective cohort study, or randomized controlled trial (RCT). The exclusion criteria encompassed 1) animal experiments; 2) review, meta-analysis, case report, comment, letter, and conference; 3) unavailable full texts; 4) written not in English; and 5) studies related to pathological mechanisms. During the initial screening, titles and abstracts of the retrieved studies were evaluated. The full text was subsequently read to determine eligibility. Disagreements were tackled through discussion with the third author (ZJ).

### 2.3 Data extraction

Information like the author, year, country, study design, institution, study period, populations, age, body mass index (BMI), FIGO stage, grade, histology, risk stratification, operation approach, and SLNB procedure were extracted from each eligible study. Two reviewers (ZS and ZDD) extracted the data and negotiated with the third author (ZJ) in case of disputes.

### 2.4 Quality assessment

Study quality was appraised via the Newcastle-Ottawa Scale (NOS) tool (21). Studies with > 6 scores were grated as high quality, while those with ≤5 scores were grated as moderate quality.

### 2.5 Statistical analysis

Heterogeneity was appraised with Cochran’s q-test and I² statistics. Data were pooled via a random-effects model (DerSimonia-Laird method) if I² >50% or P<0.10; otherwise, a fixed-effects model (Mantel-Haenszel method) was selected. Sensitivity analysis was implemented to determine the stability of the result. Egger’s test was conducted to explore publication bias. P<0.05 (two-sided) implied statistical differences. All data were analyzed by STATA18.

## 3 Results

### 3.1 Search results

Through database searches, 2,420 articles were identified. Of them, 1513 articles remained after duplicates were removed. Next, 60 articles remained following title and abstract screening. 15 articles were eligible after full-text reading, and two were excluded for indefinite hazard ratio (HR). The screening process is displayed in **Supplementary Figure S1**.

### 3.2 Study characteristics

The included studies were all retrospective cohort studies. Patients were aged from 50 to 92 years in the SLNB group and 38 to 90 years in the LND group. BMI ranged from 17.6 to 56.1 kg/m^2^ in the SLNB group and 17.2 to 60.3 kg/m^2^ in the LND group. Overall, 36,621 EC patients were included, with 10976 (30.0%) in the SLNB group and 25645 (70.0%) in the LND group. Detailed traits of the 13 studies (22–34) are summarized in **Supplementary Table S2**.

### 3.3 Quality assessment

The NOS tool indicated that all included studies had a quality score of >6, reflecting moderate to high methodological quality. All studies were retrospective in design (**Supplementary Table S3**). While most demonstrated adequate follow-up and clear outcome assessment, there were differences in the ascertainment of exposure and comparability of cohorts, indicating methodological heterogeneity and lack of standardization across studies.

### 3.4 Oncological outcomes

#### 3.4.1 OS

**Figure 1:**
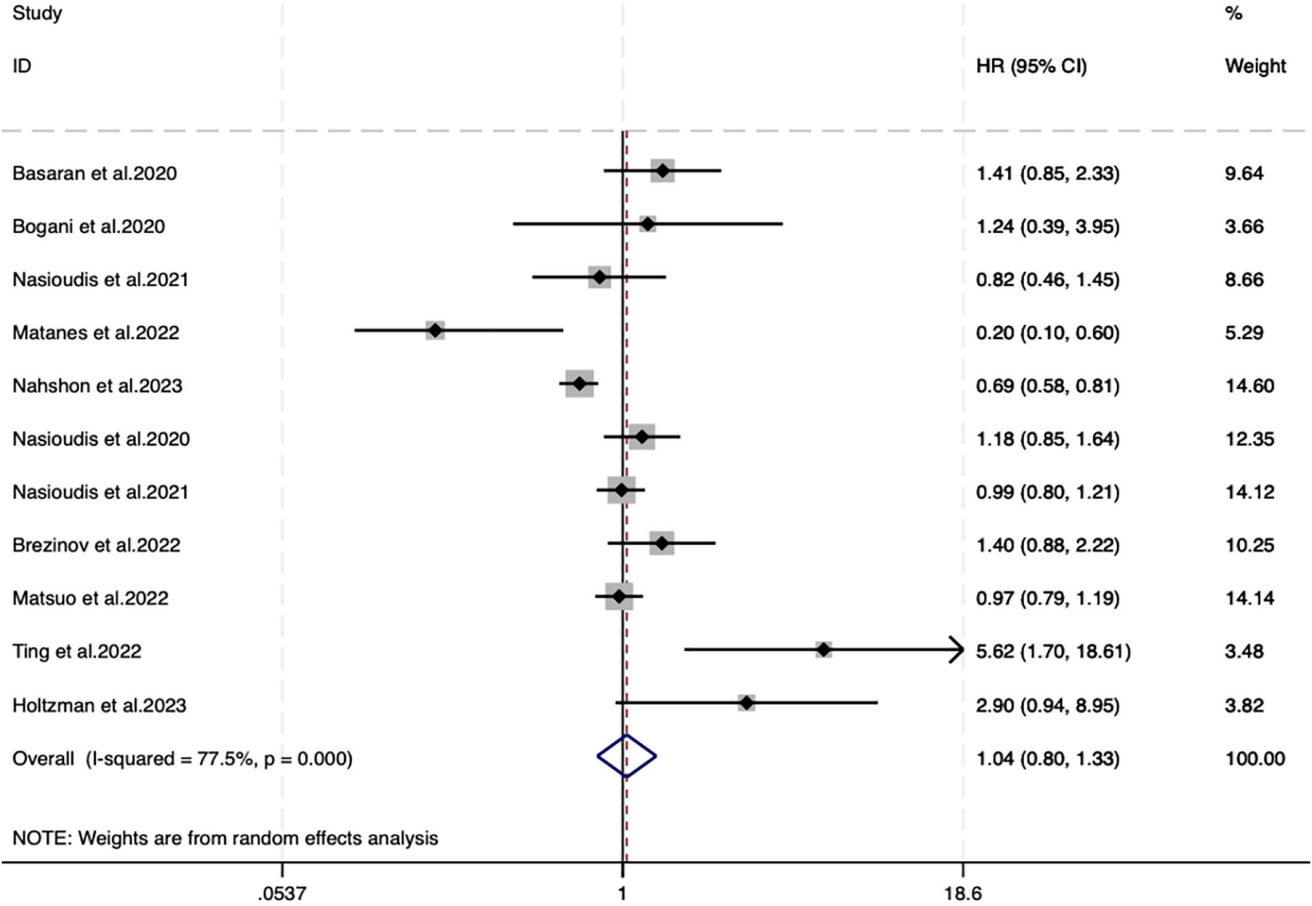
Forest of OS.

11 studies reported OS, with 10566 individuals in the SLNB group and 24905 in the LND group. A random-effects model (I^2^ = 77.5%, P<0.001) unveiled no difference in OS between SLNB and LND (HR=1.04, 95%CI: 0.80–1.33; P=0.789) (**Figure 1**).

Given high heterogeneity, subgroup analyses were designed based on risk stratification, FIGO stage, data source, sample size, and SLNB strategy. Details are manifested in Supplementary **Supplementary Figure SS2**. SLNB was not associated with OS in different continents (**Supplementary Figure S2A**. North America: HR=0.99, 95%CI: 0.75–1.29, P=0.924; Europe: HR=1.24, 95%CI: 0.39–3.95, P=0.716; Asia: HR=1.44, 95%CI: 0.62–3.37, P=0.400), FIGO stages (Supplementary **Supplementary Figure SS2B**. I-IV: HR=1.69, 95%CI: 0.75–3.82, P=0.209; I-III: HR=0.75, 95%CI: 0.34–1.65, P=0.475; I-II: HR=1.01,95%CI: 0.88–1.15, P=0.881), sample sizes (Supplementary **Supplementary Figure SS2C**. <1160: HR=1.36, 95%CI: 0.49–3.75, P=0.555; ≥1160: HR=0.96, 95%CI: 0.78–1.18, P=0.673), and SLNB strategies (**Supplementary Figure S2D**. SLNB: HR=0.75, 95%CI: 0.46–1.22, P=0.242; SLNB+LND: HR=1.21, 95%CI: 0.94–1.57, P=0.137) (**Table 1**). Subgroup analysis of data sources uncovered that SLNB in four studies originating from the survival plot yielded worse OS (**Supplementary Figure S2E**. Curves: HR=1.63, 95% CI: 1.04-2.56, P=0.035). In the subgroup analysis of risk stratification, SLNB produced better OS in intermediate and high-risk EC patients (**Supplementary Figure S2F**. Intermediate, High: HR=0.20, 95% CI: 0.08-0.49, P<0.001) (**Table 1**).

**Table 1:** Subgroup analysis of OS.

Subgroups	NO. studies	HR	95%CI	p
Area				
North America	7	0.99	0.75–1.29	0.924
Europe	1	1.24	0.39–3.95	0.716
Asia	3	1.44	0.62–3.37	0.400
FIGO stage				
I-IV	4	1.69	(0.75–3.82)	0.209
I-III	4	0.75	(0.34–1.65)	0.475
I-II	3	1.01	(0.88–1.15)	0.881
Sample size				
<1160	5	1.36	(0.49–3.75)	0.555
≥1160	6	0.96	(0.78–1.18)	0.673
SLNB strategy				
SLNB	5	0.75	(0.46–1.22)	0.242
SLNB+LND	6	1.21	(0.94–1.57)	0.137
Data Sources				
Curves	4	1.63	(1.04-2.56)	0.035
Multivariate analysis	7	0.87	(0.67-1.14)	0.320
Risk stratification				
High	3	1.34	(0.97-1.83)	0.072
Low, Intermediate, High	1	1.24	(0.39-3.95)	0.716
NR	5	0.94	(0.71-1.25)	0.678
Intermediate, High	1	0.20	(0.08-0.49)	0.000
Low, High	1	1.40	(0.88-2.22)	0.154

#### 3.4.2 PFS

**Figure 2:**
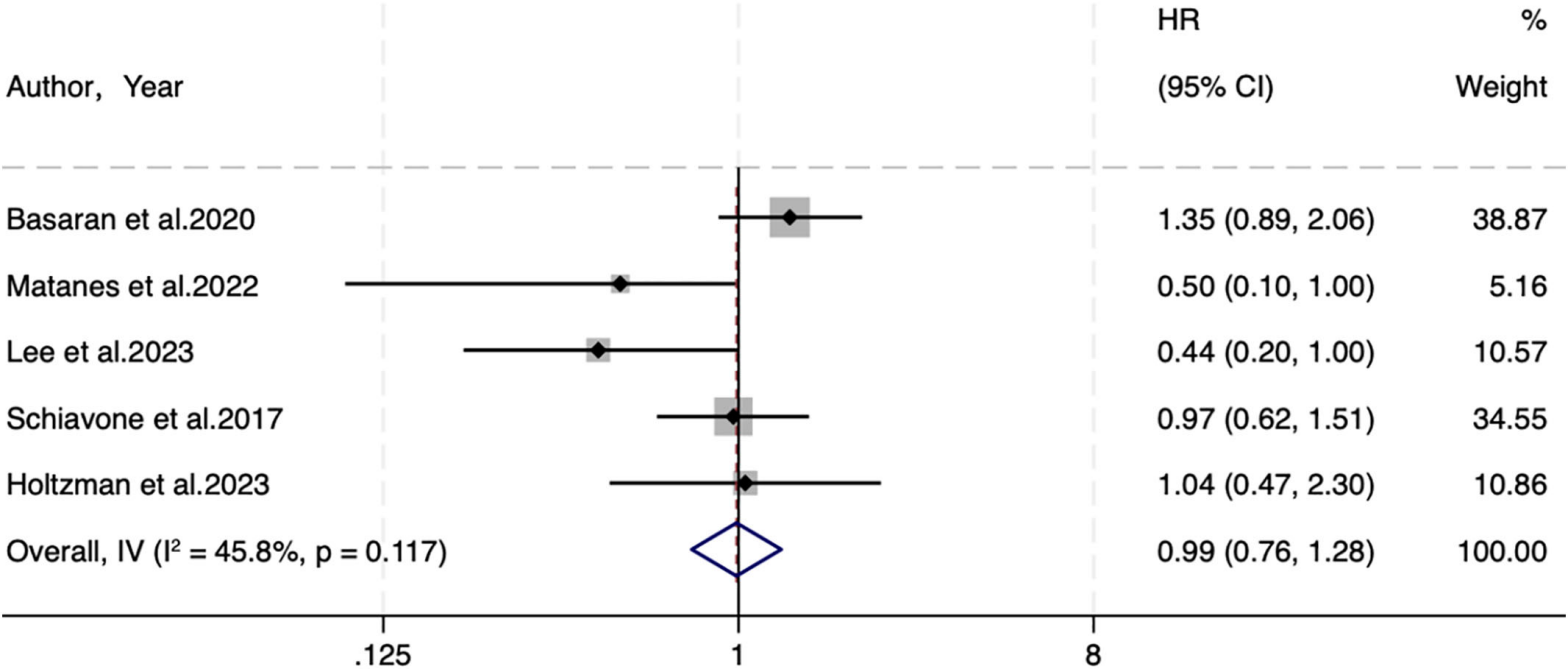
Forest of PFS.

Five studies were included regarding PFS, with 609 individuals in the SLNB group and 1,133 in the LND group. A fixed-effects model (I^2^ = 45.8%, P=0.117) elicited no statistical difference in PFS (HR=0.99, 95% CI: 0.76-1.28; P=0.927) (**Figure 2**).

Despite little heterogeneity in PFS, subgroup analyses were performed based on the same six variables described above to explore the impact of clinical traits on outcomes. Details are manifested in **Supplementary Figure S3**. SLNB was not associated with PFS in different data sources (**Supplementary Figure S3B**. Curves: HR=1.35, 95%CI: 0.89–2.05, P=0.161, multivariate analysis: HR=0.64, 95%CI: 0.39–1.06, P=0.086, univariate analysis: HR=0.97, 95%CI: 0.62–1.51, P=0.893), sample sizes (**Supplementary Figure S3C**. ≥245: HR=1.02, 95%CI: 0.77-1.36, P=0.874, <245: HR=0.82, 95%CI: 0.43–1.58, P=0.555), and SLNB strategies (**Supplementary Figure S3D**. SLNB: HR=0.99, 95%CI: 0.69-1.41, P=0.952, SLNB+LND: HR=0.99, 95%CI: 0.67-1.45, P=0.944) (**Table 2**). In the subgroup analysis of continents, 1 study in Asia suggested that SLNB yielded better PFS (**Supplementary Figure S3A**. Asia: HR=0.44, 95% CI: 0.20-0.98, P=0.04). In the subgroup analysis of risk stratification, 1 study of not reported (NR) risk showed that SLNB yielded better PFS (**Supplementary Figure S3E**. NR: HR=0.44, 95% CI: 0.20-0.98, P=0.046). In the subgroup analysis of FIGO stage, two studies on stage I-III EC demonstrated that SLNB produced better PFS (**Supplementary Figure S3F**. I-III: HR=0.46, 95% CI: 0.24-0.89, P=0.021) (**Table 2**).

**Table 2:** Subgroup analysis of PFS.

Subgroups	NO. studies	HR	95%CI	p
Area				
North America	4	1.09	(0.82–1.43)	0.555
Asia	1	0.44	(0.20–0.98)	0.046
Data Sources				
Curves	1	1.35	(0.89-2.05)	0.161
Multivariate analysis	3	0.64	(0.39-1.06)	0.086
Univariate analysis	1	0.97	(0.62-1.51)	0.893
Sample size				
≥245	3	1.02	(0.77–1.36)	0.874
<245	2	0.82	(0.43–1.58)	0.555
SLNB strategy				
SLNB	3	0.99	(0.69–1.41)	0.952
SLNB+LND	2	0.99	(0.67–1.45)	0.944
Risk stratification				
High	3	1.14	(0.86-1.52)	0.368
Intermediate, High	1	0.50	(0.16-1.58)	0.238
NR	1	0.44	0.20-0.98	0.046
FIGO stage				
I-IV	3	1.14	(0.86–1.52)	0.368
I-III	2	0.46	(0.24–0.89)	0.021

#### 3.4.3 DSS

**Figure 3:**
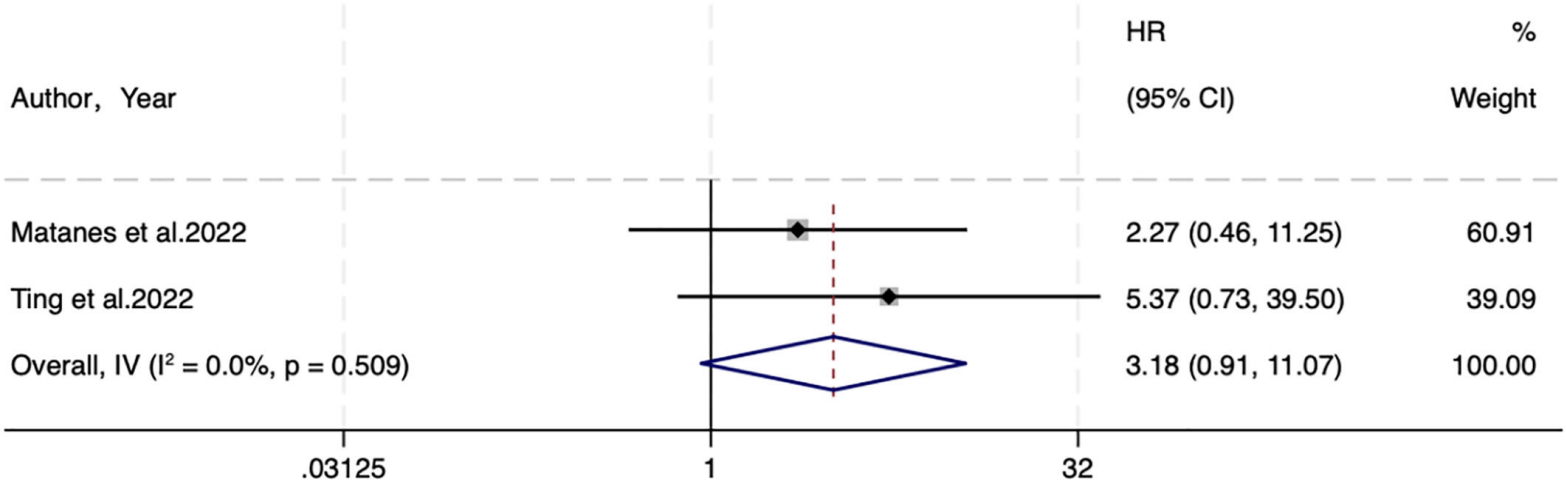
Forest of DSS.

Two studies analyzed DSS. A fixed-effects (I^2^ = 0.0%, P=0.509) analysis elicited no statistical difference between SLNB and LND in DSS (HR=3.18, 95% CI: 0.91-11.07; P=0.069) (**Figure 3**).

### 3.5 Sensitivity analysis and publication bias

**Figure 4:**
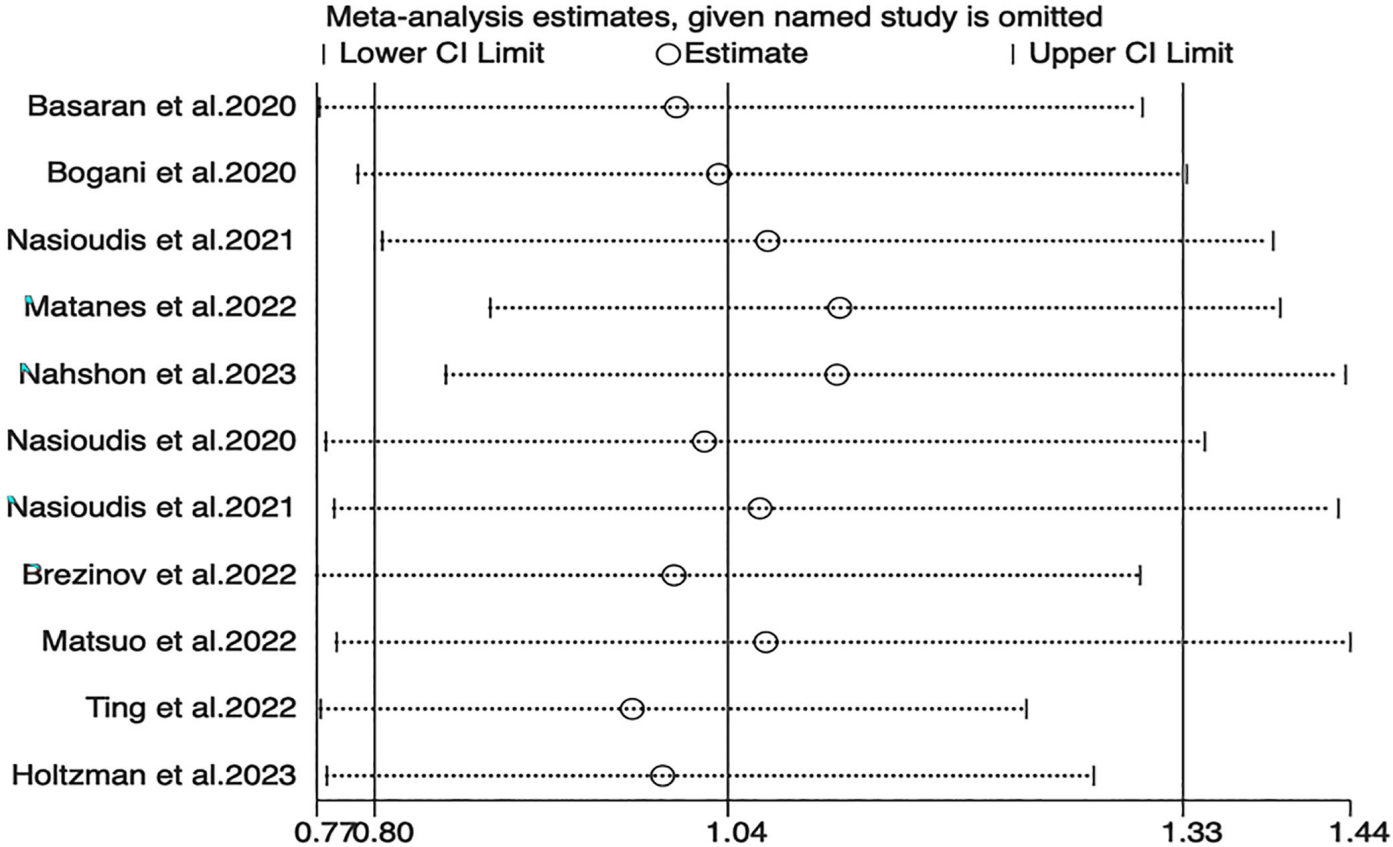
Sensitivity analysis of OS.

**Figure 5:**
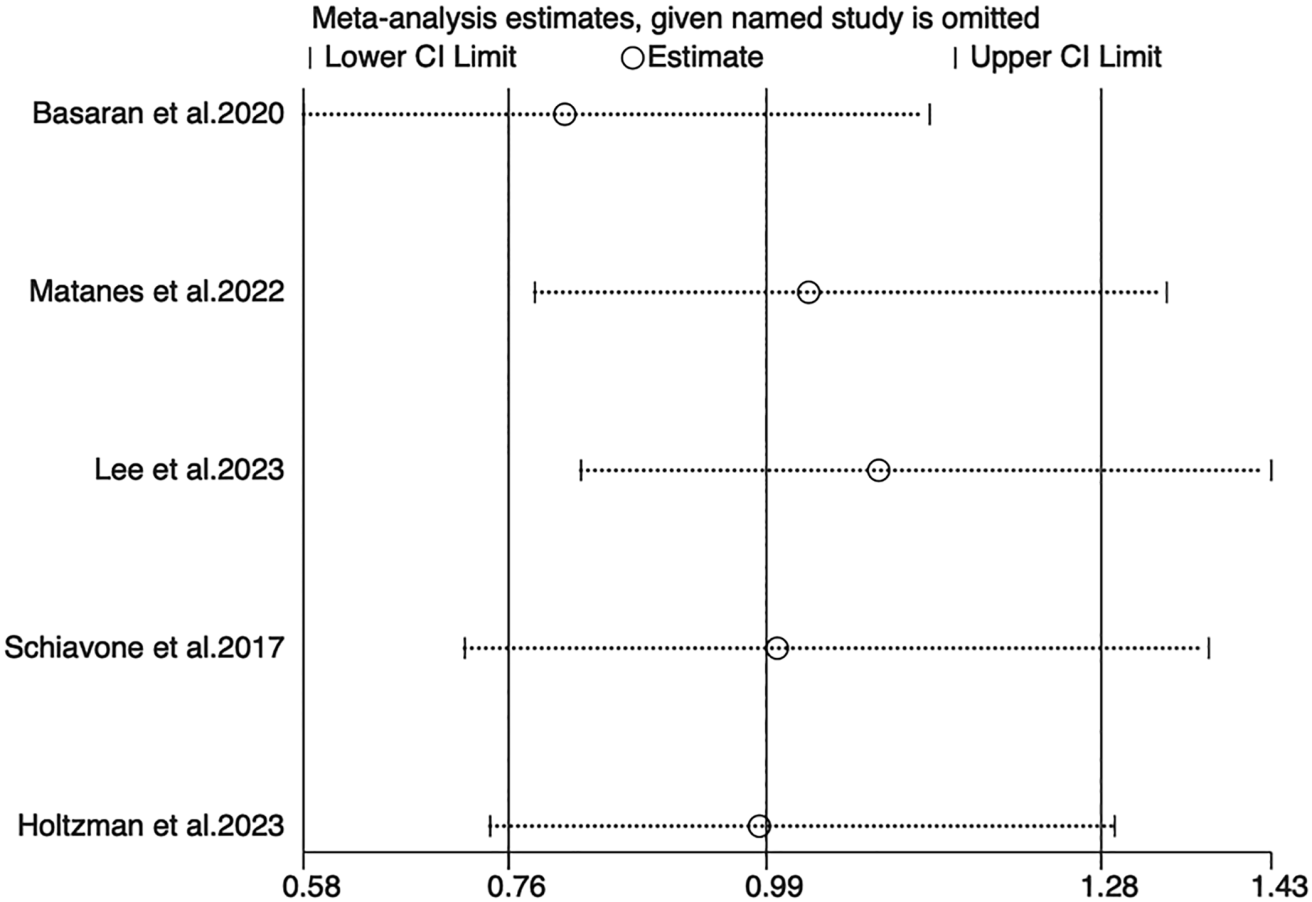
Sensitivity analysis of PFS.

**Figure 6:**
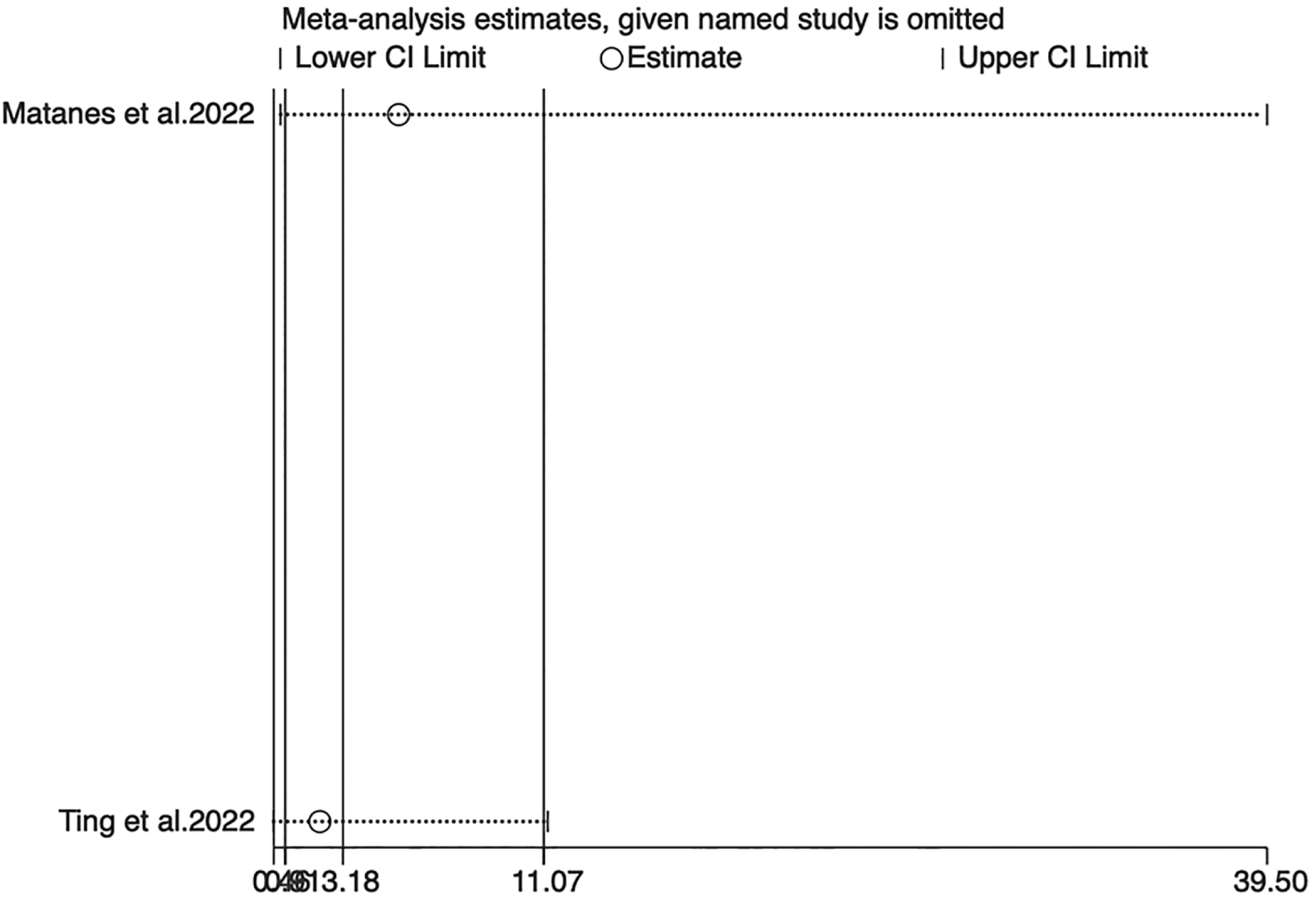
Sensitivity analysis of DSS.

Sensitivity analyses for OS, PFS, and DSS found that the merged results were all relatively stable (**Figures 4**–**6**). Egger’s test indicated no publication bias for OS (P=0.211).

## 4 Discussion

This meta-analysis combined and analyzed all studies that compared SLNB and LND in the prognostic impact on EC patients. The results illustrated no difference in the prognosis of EC patients between SLNB and LND. However, the OS subgroup analysis suggested that SLNB from survival graphs had worse OS than that of studies that provided original HR data, and intermediate and high-risk EC had better OS; and the PFS subgroup analysis suggested that SLNB in the studies of stage I-III EC and the studies in Asia yielded better PFS.

Similar to Gu, Y et al. (35), the present study pooled available comparisons of SLNB and LND in EC and noticed no differences in OS, PFS, and DSS between these two approaches. The survival HRs were analyzed in this study, whereas Gu, Y et al. compared survival rates from only three publications, with a follow-up period of survival ranging from 3 to 6 years, which could contribute to different results to some extent. In addition, subgroup analyses were performed based on the continent, risk stratification, FIGO stage, data source, sample size, and SLNB strategy. The results elicited that the conclusions were influenced by risk stratification and FIGO stage. Additionally, in previous studies (36, 37), some patients in both the SLNB and LND groups received additional measures at a later stage, which may have influenced outcomes in both groups. For a more rigorous analysis, we restricted the LND group to individuals who did not receive SLNB. In existing studies, patients in the SLNB group often received preparatory or elective LND after SLNB failure, resulting in fewer SLNB-only patients. Therefore, the inclusion criteria for the SLNB cohort in this study were broader, and participants in the SLNB group receiving LND were also included. However, this meta-analysis analyzed SLNB as an independent surgical strategy in a separate subgroup, thus presenting more accurate results.

The main treatment regimen for EC is surgery, and a joint announcement by the American College of Obstetricians and Gynecologists and the Society of Gynecologic Oncology recommends surgical staging in initial treatment (38). LND is currently the main modality for determining LNM (39), but the clinical value of LND is highly controversial (40). Some articles have shown that up to 90% of stage I EC patients receive non-essential LND (41). Recent ESGO/ESTRO/ESP guidelines and other guidelines recommend SLNB for standardized clinical treatment of EC but mainly focus on early-stage low-risk EC patients (42, 43)). Few studies focus on intermediate and high-risk cohorts, and the application and effectiveness of SLNB for high-risk EC patients remain significantly controversial (44, 45). However, available studies have shown that in patients with intermediate- to high-risk EC, SLNB can almost completely exclude lymph node metastasis when performed by experienced physicians. Although no relevant studies on long-term survival have been conducted, existing evidence supports that SLNB can replace LND in intermediate- and high-risk EC (46).

In this meta-analysis, four articles included focused on high-risk EC. Subgroup analysis unveiled no considerable differences in OS and PFS between SLNB and LND in high-risk EC. However, these findings are based on a limited number of studies. More studies are needed to assess the oncological safety and prognostic value of SLNB in this specific patient population (37). In intermediate-high EC, SLNB had better OS. However, the findings were only from an article on older adults, and the conclusions need to be interpreted with caution. More investigations are warranted to ascertain prognostic differences between SLNB and LND for intermediate- and high-risk EC populations. Overall, SLNB yields either indistinguishable or better outcomes than LND, suggesting that SLNB is applicable regardless of the population. In addition to long-term survival outcomes, SLNB offers significant advantages in reducing postoperative dysfunction. Studies have shown that SLNB significantly reduces the incidence of lower-extremity lymphedema, which in turn improves patients’ quality of life (46). Compared with systematic lymph node dissection, SLNB preserves postoperative quality of life while maintaining tumor control. Furthermore, one of the key advantages of SLNB lies in its ability to perform ultrastaging, thus enhancing the detection of low-volume metastases such as micrometastases and isolated tumor cells (ITCs), which are often missed with conventional lymphadenectomy. A recent multicenter study of low-risk EC patients who underwent SLNB without adjuvant therapy demonstrated that the presence of ITCs in SLNs was associated with significantly worse recurrence-free survival compared to node-negative patients, despite similar clinicopathologic features (47). The evidence helps explain the comparable or even improved outcomes observed in patients undergoing SLNB.

Confounding factors in multivariate analyses were also analyzed. Eight studies assessed prognostic outcomes through multivariate analyses, and these factors included age, ASA score, tumor stage, grade, lymphovascular space invasion presence, adjuvant therapy, and surgical approach. In one of the studies (27), before multivariate correction, notable differences occurred in uncorrected OS between SLNB and LND, with LND having better OS. After adjustment for age, adjuvant therapy, and surgical approach, no significant difference in oncologic outcomes was revealed between the two groups. In another article (18), even after adjustment for ASA score, age, tumor grade, tumor stage, and lymphovascular space invasion presence, SLNB had better OS. Those findings confirm the potential impact of adjuvant therapy and surgical approach on EC prognosis, which should be substantiated in future research. Although molecular typing was not evaluated in the present meta-analysis, data from the latest study suggest that molecular typing is valuable in the prognostic stratification of EC patients with lymph node metastasis. The results of a multicenter study showed that p53-abnormal and mismatch repair-deficient types were associated with different risks of recurrence in lymph node-positive patients (48). However, differences in SLNB mapping techniques, such as blue dye versus indocyanine green (ICG) fluorescence, may influence detection rates and oncologic outcomes. Previous research suggests that ICG fluorescence improves the detection rate of sentinel lymph nodes compared to blue dye alone (49), which could impact survival outcomes and false-negative rates. Future studies should standardize SLNB methodologies to minimize discrepancies across studies.

There are also certain limitations. First, most articles included are retrospective cohort studies, which prevents high-grade assessment. Future prospective RCTs are warranted to compare SLNB and LND. Second, some included studies only provide survival curves, and statistical methods are used to acquire proper data, which may increase inaccuracy. Future studies should ensure that HRs and confidence intervals are explicitly reported to enhance data reliability. Third, although the subgroup analysis of risk stratification was performed, there are few articles examining high/moderate-high risk, limiting the interpretation of the results.

## 5 Conclusion

This paper pooled current evidence on the comparisons of SLNB and LND and illustrated that SLNB did not reduce survival in EC patients. However, in intermediate and high-risk EC, SLNB yielded better OS. This conclusion may be restrained by the number of articles. More prospective studies or large RCTs are warranted to further substantiate the conclusion in intermediate and high-risk EC.

## Data Availability

The original contributions presented in the study are included in the article/**Supplementary Material**. Further inquiries can be directed to the corresponding author.

## References

[1] Barczynski B Fraszczak K Bednarek W . Sentinel lymph node mapping in endometrial cancer after 2020 esgo-estro-esp consensus update: what will happen in the next few years? Ginekol Pol. (2022) 93:321–8. doi: 10.5603/GP.a2021.0214 35156700

[2] Sung H Ferlay J Siegel RL Laversanne M Soerjomataram I Jemal A . Global cancer statistics 2020: globocan estimates of incidence and mortality worldwide for 36 cancers in 185 countries. CA Cancer J Clin. (2021) 71:209–49. doi: 10.3322/caac.21660 33538338

[3] Lewin SN Herzog TJ Barrena Medel NI Deutsch I Burke WM Sun X . Comparative performance of the 2009 international federation of gynecology and obstetrics’ Staging system for uterine corpus cancer. Obstet Gynecol. (2010) 116:1141–9. doi: 10.1097/AOG.0b013e3181f39849 20966700

[4] Peters EEM León-Castillo A Smit V Boennelycke M Hogdall E Hogdall C . Defining substantial lymphovascular space invasion in endometrial cancer. Int J Gynecol Pathol. (2022) 41:220–6. doi: 10.1097/pgp.0000000000000806 34261899

[5] Clark LH Soper JT . Endometrial cancer and the role of lymphadenectomy. Obstet Gynecol Surv. (2016) 71:353–60. doi: 10.1097/ogx.0000000000000321 27302186

[6] Baiocchi G Andrade C Ribeiro R Moretti-Marques R Tsunoda AT Alvarenga-Bezerra V . Sentinel lymph node mapping versus sentinel lymph node mapping with systematic lymphadenectomy in endometrial cancer: an open-label, non-inferiority, randomized trial (Alice trial). Int J Gynecol Cancer. (2022) 32:676–9. doi: 10.1136/ijgc-2022-003378 35236752

[7] Abu-Rustum N Yashar C Arend R Barber E Bradley K Brooks R . Uterine neoplasms, version 1.2023, nccn clinical practice guidelines in oncology. J Natl Compr Canc Netw. (2023) 21:181–209. doi: 10.6004/jnccn.2023.0006 36791750

[8] Pecorelli S . Revised figo staging for carcinoma of the vulva, cervix, and endometrium. Int J Gynaecol Obstet. (2009) 105:103–4. doi: 10.1016/j.ijgo.2009.02.012 19367689

[9] Benedetti Panici P Basile S Maneschi F Alberto Lissoni A Signorelli M Scambia G . Systematic pelvic lymphadenectomy vs. No lymphadenectomy in early-stage endometrial carcinoma: randomized clinical trial. J Natl Cancer Inst. (2008) 100:1707–16. doi: 10.1093/jnci/djn397 19033573

[10] Helgers RJA Winkens B Slangen BFM Werner HMJ . Lymphedema and post-operative complications after sentinel lymph node biopsy versus lymphadenectomy in endometrial carcinomas-a systematic review and meta-analysis. J Clin Med. (2020) 10:20–136. doi: 10.3390/jcm10010120 PMC7795280 33396373

[11] Joly F McAlpine J Nout R Åvall-Lundqvist E Shash E Friedlander M . Quality of life and patient-reported outcomes in endometrial cancer clinical trials: A call for action! Int J Gynecol Cancer. (2014) 24:1693–9. doi: 10.1097/igc.0000000000000299 25340294

[12] Kang S Yoo HJ Hwang JH Lim MC Seo SS Park SY . Sentinel lymph node biopsy in endometrial cancer: meta-analysis of 26 studies. Gynecol Oncol. (2011) 123:522–7. doi: 10.1016/j.ygyno.2011.08.034 21945553

[13] Farzaneh F Moridi A Azizmohammadi Z Ansari JM Hosseini MS Arab M . Value of sentinel lymph node (Sln) mapping and biopsy using combined intracervical radiotracers and blue dye injections for endometrial cancer. Asian Pac J Cancer Prev. (2017) 18:431–5. doi: 10.22034/apjcp.2017.18.2.431 PMC5454739 28345826

[14] Bedyńska M Szewczyk G Klepacka T SaChadel K Maciejewski T Szukiewicz D . Sentinel lymph node mapping using indocyanine green in patients with uterine and cervical neoplasms: restrictions of the method. Arch Gynecol Obstet. (2019) 299:1373–84. doi: 10.1007/s00404-019-05063-6 PMC6475506 30762108

[15] Concin N Matias-Guiu X Vergote I Cibula D Mirza MR Marnitz S . Esgo/estro/esp guidelines for the management of patients with endometrial carcinoma. Int J Gynecol Cancer. (2021) 31:12–39. doi: 10.1136/ijgc-2020-002230 33397713

[16] Baiocchi G Mantoan H Kumagai LY Gonçalves BT Badiglian-Filho L de Oliveira Menezes AN . The impact of sentinel node-mapping in staging high-risk endometrial cancer. Ann Surg Oncol. (2017) 24:3981–7. doi: 10.1245/s10434-017-6132-8 29058141

[17] Buda A Di Martino G Restaino S De Ponti E Monterossi G Giuliani D . The impact on survival of two different staging strategies in apparent early stage endometrial cancer comparing sentinel lymph nodes mapping algorithm and selective lymphadenectomy: an italian retrospective analysis of two reference centers. Gynecol Oncol. (2017) 147:528–34. doi: 10.1016/j.ygyno.2017.09.033 29032824

[18] Ignatov A Papathemelis T Ivros S Ignatov T Ortmann O Eggemann H . Comparison of survival of patients with endometrial cancer undergoing sentinel node biopsy alone or systematic lymphadenectomy. Arch Gynecol Obstet. (2020) 302:995–1000. doi: 10.1007/s00404-020-05698-w 32666131

[19] Schlappe BA Weaver AL McGree ME Ducie J Zahl Eriksson AG Dowdy SC . Multicenter study comparing oncologic outcomes after lymph node assessment via a sentinel lymph node algorithm versus comprehensive pelvic and paraaortic lymphadenectomy in patients with serous and clear cell endometrial carcinoma. Gynecol Oncol. (2020) 156:62–9. doi: 10.1016/j.ygyno.2019.11.002 PMC6980738 31776037

[20] Vrabel M . Preferred reporting items for systematic reviews and meta-analyses. Oncol Nurs Forum. (2015) 42:552–4. doi: 10.1188/15.Onf.552-554 26302284

[21] Wells GA Shea B O’Connell D Peterson J Welch V Losos M . The newcastle-ottawa scale (Nos) for assessing the quality of nonrandomised studies in meta-analyses (2021). Available online at: https://www.ohri.ca/programs/clinical_epidemiology/oxford.asp (Accessed January 20, 2024).

[22] Basaran D Bruce S Aviki EM Mueller JJ Broach VA Cadoo K . Sentinel lymph node mapping alone compared to more extensive lymphadenectomy in patients with uterine serous carcinoma. Gynecol Oncol. (2020) 156:70–6. doi: 10.1016/j.ygyno.2019.10.005 PMC6980657 31739992

[23] Bogani G Casarin J Leone Roberti Maggiore U Ditto A Pinelli C Dell’acqua A . Survival outcomes in endometrial cancer patients having lymphadenectomy, sentinel node mapping followed by lymphadectomy and sentinel node mapping alone: long-term results of a propensity-matched analysis. Gynecol Oncol. (2020) 158:77–83. doi: 10.1016/j.ygyno.2020.04.691 32389376

[24] Nasioudis D Byrne M Ko EM Giuntoli Ii RL Haggerty AF Cory L . The impact of sentinel lymph node sampling versus traditional lymphadenectomy on the survival of patients with stage iiic endometrial cancer. Int J Gynecol Cancer. (2021) 31:840–5. doi: 10.1136/ijgc-2021-002450 33853879

[25] Matanes E Eisenberg N Mitric C Yasmeen A Ismail S Raban O . Surgical and oncological outcomes of sentinel lymph node sampling in elderly patients with intermediate to high-risk endometrial carcinoma. Int J Gynecol Cancer. (2022) 32:875–81. doi: 10.1136/ijgc-2022-003431 35680137

[26] Lee D Kim YN Cho SH Yoon HS Kim YT Kim S . Comparison of progression-free survival outcome of sentinel node biopsy without ultrastaging versus lymphadenectomy in endometrial cancer: A propensity-matched analysis. J Gynecol Oncol. (2023) 34:e79. doi: 10.3802/jgo.2023.34.e79 37477102 PMC10627756

[27] Nahshon C Kadan Y Lavie O Ostrovsky L Segev Y . Sentinel lymph node sampling versus full lymphadenectomy in endometrial cancer: A seer database analysis. Int J Gynecol Cancer. (2023) 33:1557–63. doi: 10.1136/ijgc-2023-004474 37487660

[28] Schiavone MB Scelzo C Straight C Zhou Q Alektiar KM Makker V . Survival of patients with serous uterine carcinoma undergoing sentinel lymph node mapping. Ann Surg Oncol. (2017) 24:1965–71. doi: 10.1245/s10434-017-5816-4 PMC6092025 28258415

[29] Nasioudis D Albright BB Roy A Ko EM Giuntoli RL 2nd Haggerty AF . Patterns of use and outcomes of sentinel lymph node mapping for patients with high-grade endometrial cancer. Gynecol Oncol. (2020) 159:732–6. doi: 10.1016/j.ygyno.2020.09.023 33004215

[30] Nasioudis D Byrne M Ko EM Giuntoli RL 2nd Haggerty AF Cory L . Outcomes of sentinel lymph node mapping for patients with figo stage I endometrioid endometrial carcinoma. Gynecol Oncol. (2021) 161:705–9. doi: 10.1016/j.ygyno.2021.03.018 33771397

[31] Brezinov Y Katzir T Gemer O Helpman L Eitan R Vaknin Z . Does sentinel lymph node biopsy in endometrial cancer surgery have an impact on the rate of adjuvant post operative pelvic radiation? An Israeli gynecologic oncology group study. Gynecol Oncol Rep. (2022) 41:100978. doi: 10.1016/j.gore.2022.100978 35469128 PMC9034297

[32] Matsuo K Klar M Khetan VU Violette CJ Nusbaum DJ Muderspach LI . Sentinel lymph node biopsy for stage ii endometrial cancer: recent utilization and outcome in the United States. Gynecol Oncol. (2022) 164:46–52. doi: 10.1016/j.ygyno.2021.10.085 34728108

[33] Ting WH Hsieh SW Chen HH Wei MC Lin HH Hsiao SM . Predictors for the recurrence of clinically uterine-confined endometrial cancer and the role of cytokeratin immunohistochemistry stain in the era of sentinel lymph node mapping. Cancers (Basel). (2022) 14:1973–86. doi: 10.3390/cancers14081973 PMC9031387 35454878

[34] Holtzman S Stoffels G Flint M Carr C Prasad-Hayes M Zeligs K . Outcomes for patients with high-risk endometrial cancer undergoing sentinel lymph node assessment versus full lymphadenectomy. Gynecol Oncol. (2023) 174:273–7. doi: 10.1016/j.ygyno.2023.05.002 37270906

[35] Gu Y Cheng H Zong L Kong Y Xiang Y . Operative and oncological outcomes comparing sentinel node mapping and systematic lymphadenectomy in endometrial cancer staging: meta-analysis with trial sequential analysis. Front Oncol. (2020) 10:580128. doi: 10.3389/fonc.2020.580128 33520696 PMC7838488

[36] Matanes E Eisenberg N Amajoud Z Gupta V Yasmeen A Ismail S . Sentinel lymph node sampling as an alternative to lymphadenectomy in patients with endometrial cancer and obesity. J Obstet Gynaecol Can. (2021) 43:1136–44.e1. doi: 10.1016/j.jogc.2021.04.014 33984524

[37] Capozzi VA Rosati A Maglietta G Vargiu V Scarpelli E Cosentino F . Long-term survival outcomes in high-risk endometrial cancer patients undergoing sentinel lymph node biopsy alone versus lymphadenectomy. Int J Gynecol Cancer. (2023) 33:1013–20. doi: 10.1136/ijgc-2023-004314 37068852

[38] American College of Obstetricians and Gynecologists (ACOG) . Practice bulletin no. 149: endometrial cancer. Obstet Gynecol. (2015) 125:1006–26. doi: 10.1097/01.AOG.0000462977.61229.de 25798986

[39] Bogani G Mariani A Paolini B Ditto A Raspagliesi F . Low-volume disease in endometrial cancer: the role of micrometastasis and isolated tumor cells. Gynecol Oncol. (2019) 153:670–5. doi: 10.1016/j.ygyno.2019.02.027 30833134

[40] Kitchener H Swart AM Qian Q Amos C Parmar MK . Efficacy of systematic pelvic lymphadenectomy in endometrial cancer (Mrc astec trial): A randomised study. Lancet. (2009) 373:125–36. doi: 10.1016/s0140-6736(08)61766-3 PMC2646126 19070889

[41] Milam MR Java J Walker JL Metzinger DS Parker LP Coleman RL . Nodal metastasis risk in endometrioid endometrial cancer. Obstet Gynecol. (2012) 119:286–92. doi: 10.1097/AOG.0b013e318240de51 PMC3266531 22270280

[42] Colombo N Creutzberg C Amant F Bosse T González-Martín A Ledermann J . Esmo-esgo-estro consensus conference on endometrial cancer: diagnosis, treatment and follow-up. Int J Gynecol Cancer. (2016) 26:2–30. doi: 10.1097/igc.0000000000000609 26645990 PMC4679344

[43] Ballester M Dubernard G Lécuru F Heitz D Mathevet P Marret H . Detection rate and diagnostic accuracy of sentinel-node biopsy in early stage endometrial cancer: A prospective multicentre study (Senti-endo). Lancet Oncol. (2011) 12:469–76. doi: 10.1016/s1470-2045(11)70070-5 21489874

[44] Chinese Society of Research Hospitals obstetrics and Gynecology professional committee . Expert consensus on the clinical application of sentinel lymph node dissection in endometrial cancer. Chin J Clin Obstetrics Gynecology. (2020) 21:438–40. doi: 10.13390/j.issn.1672-1861.2020.04.033

[45] Bhatla N Denny L . Figo cancer report 2018. Int J Gynaecol Obstet. (2018) 143 Suppl 2:2–3. doi: 10.1002/ijgo.12608 30306587

[46] Leitao MM Jr. Zhou QC Gomez-Hidalgo NR Iasonos A Baser R Mezzancello M . Patient-Reported Outcomes after Surgery for Endometrial Carcinoma: Prevalence of Lower-Extremity Lymphedema after Sentinel Lymph Node Mapping Versus Lymphadenectomy. Gynecol Oncol. (2020) 156:147–53. doi: 10.1016/j.ygyno.2019.11.003 PMC6980687 31780238

[47] Cucinella G Schivardi G Zhou XC AlHilli M Wallace S Wohlmuth C . Prognostic value of isolated tumor cells in sentinel lymph nodes in low risk endometrial cancer: results from an international multi-institutional study. Int J Gynecol Cancer. (2024) 34:179–87. doi: 10.1136/ijgc-2023-005032 38088182

[48] Schivardi G Caruso G De Vitis LA Cucinella G Multinu F Zanagnolo V . Impact of molecular classification on recurrence risk in endometrial cancer patients with lymph node metastasis: multicenter retrospective study. Int J Gynecol Cancer. (2024) 34:1561–9. doi: 10.1136/ijgc-2024-005672 39153830

[49] Chacon E El Hajj H Hsu HC Bizzarri N Tsibulak I Collins A . Global practice patterns of sentinel lymph node biopsy in endometrial cancer: A survey from the european network of young gynecologic oncologists (Enygo). Int J Gynecol Cancer. (2024):100068. doi: 10.1016/j.ijgc.2024.100068 39955189

