# Supplementary material for: Long-term outcomes in patients with endometrial cancer after sentinel lymph node biopsy versus lymphadenectomy alone: a meta-analysis
Source: Front Oncol. 2025 May 20;15:1584447. doi: 10.3389/fonc.2025.1584447 (PMC12130034; doi:10.3389/fonc.2025.1584447)
Supplement: Supplementary file 1 [file DataSheet1.docx]

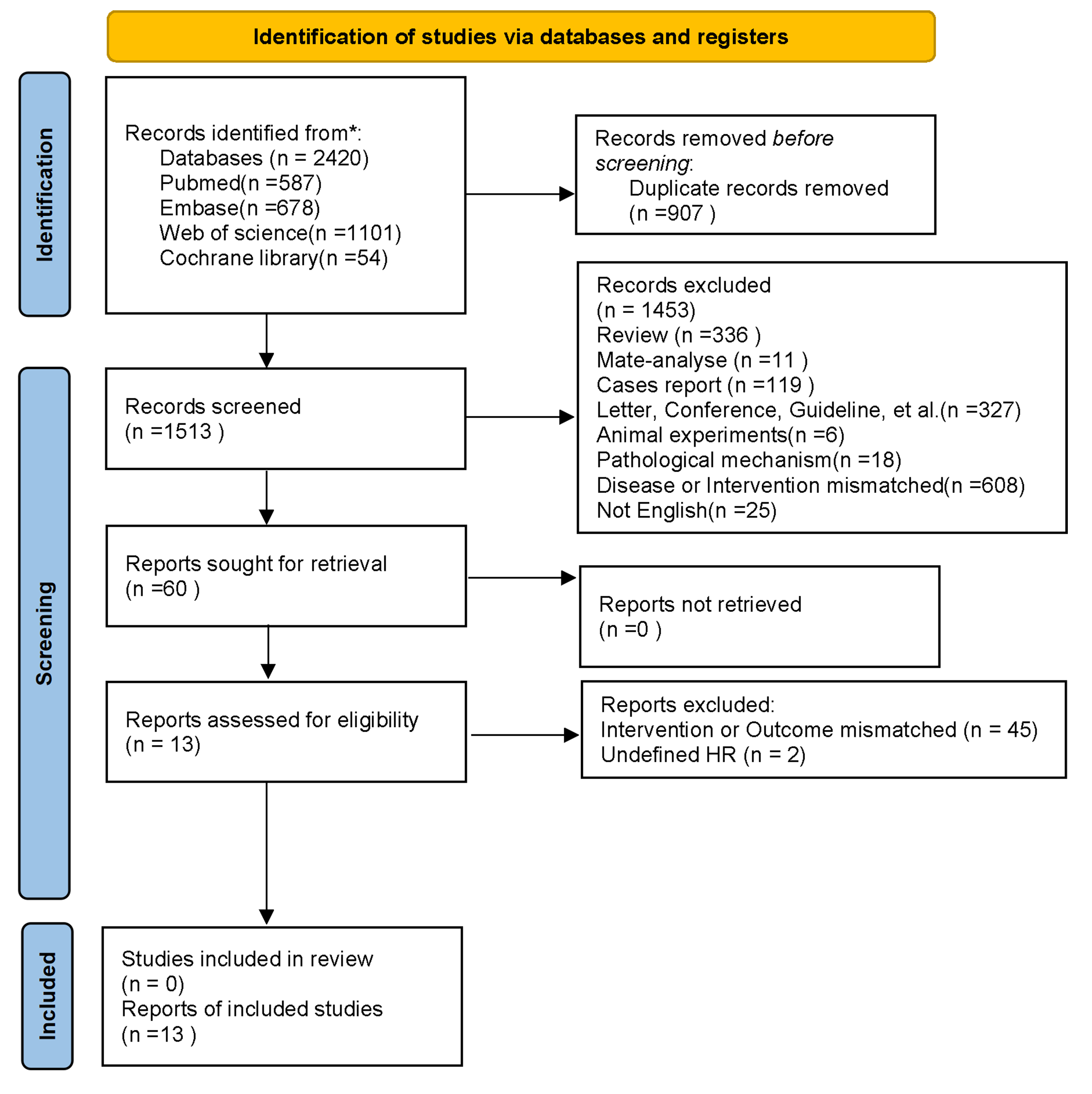


**Supplementary Figure 1.** Screening process

a. b.


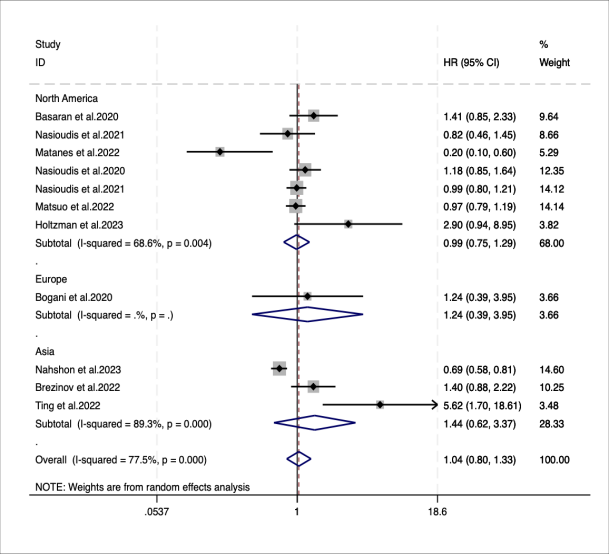

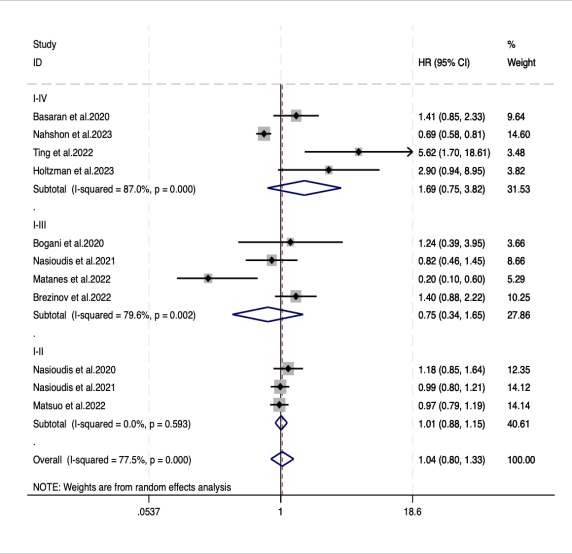


c. d.


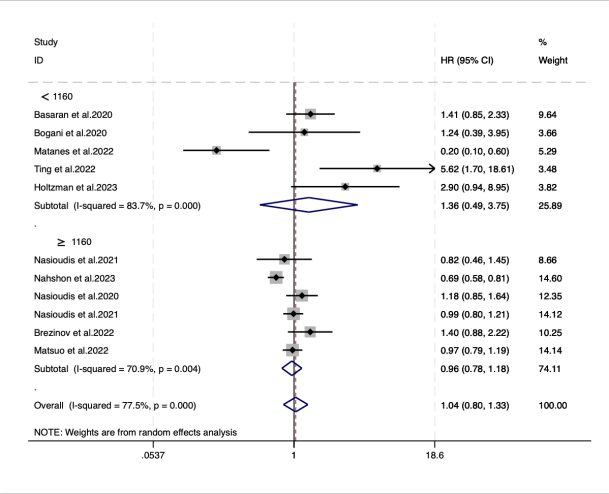

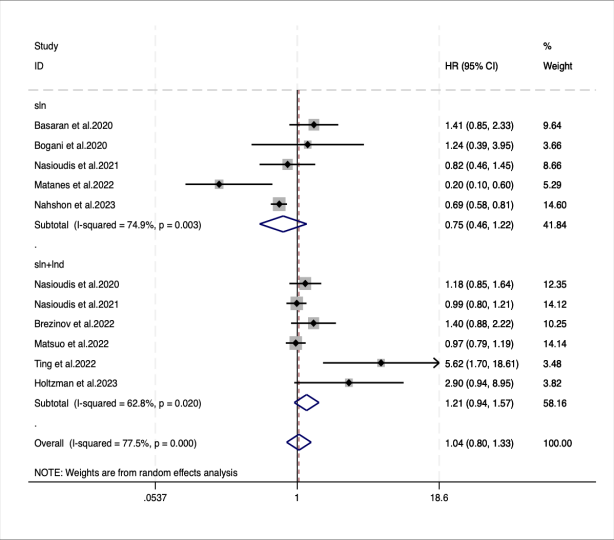


e. f.


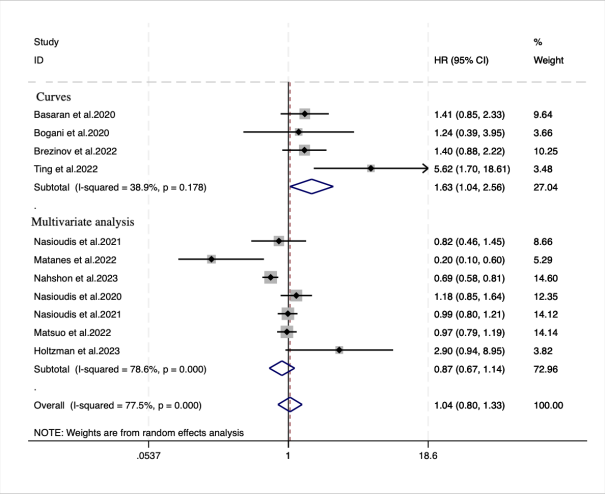

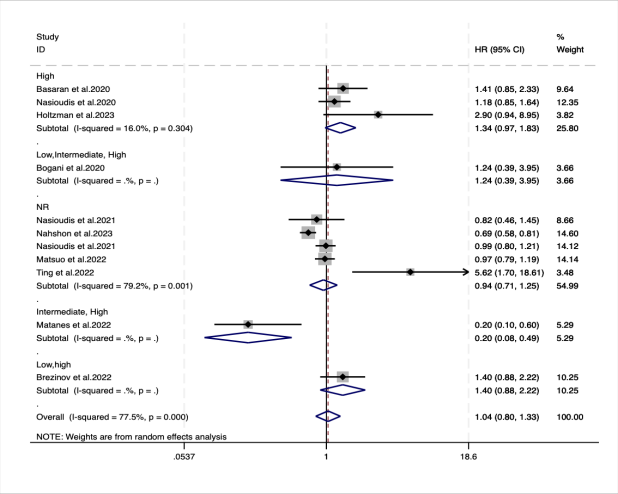


**Supplementary Figure 2. a.** Subgroup analysis of OS by continents**; b.** Subgroup analysis of OS by FIGO stage**; c.** Subgroup analysis of OS by sample size**; d.** Subgroup analysis of OS by SLNB strategy**; e.** Subgroup analysis of OS by data sources**; f.** Subgroup analysis of OS by risk stratification

a. b.


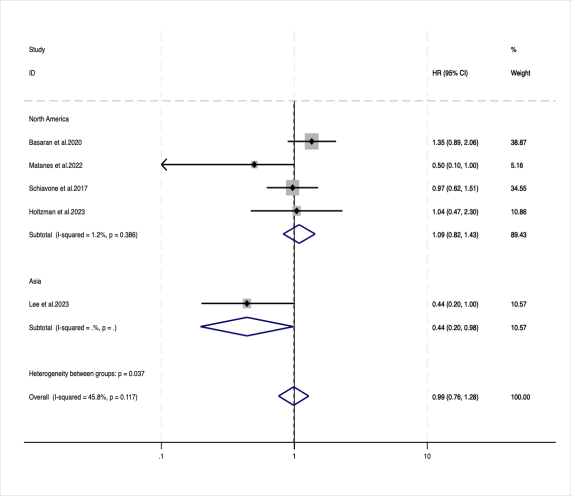

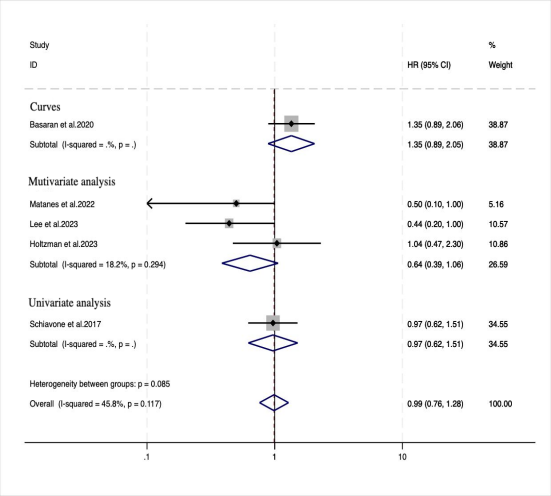


c. d.


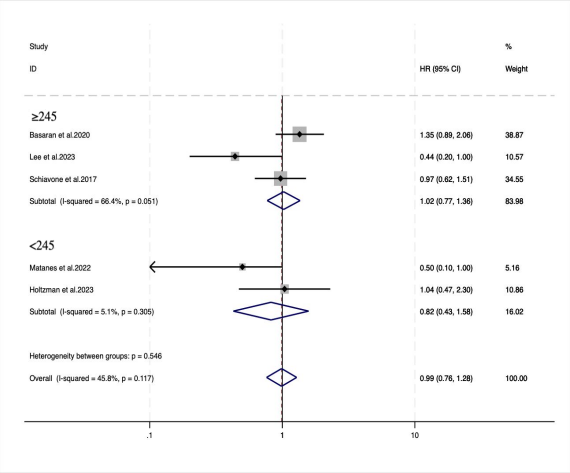

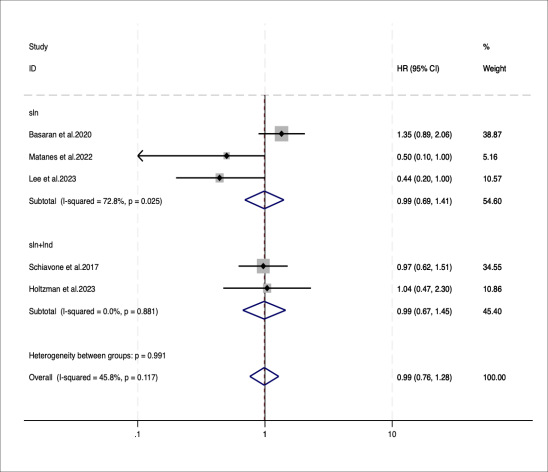


e. f.


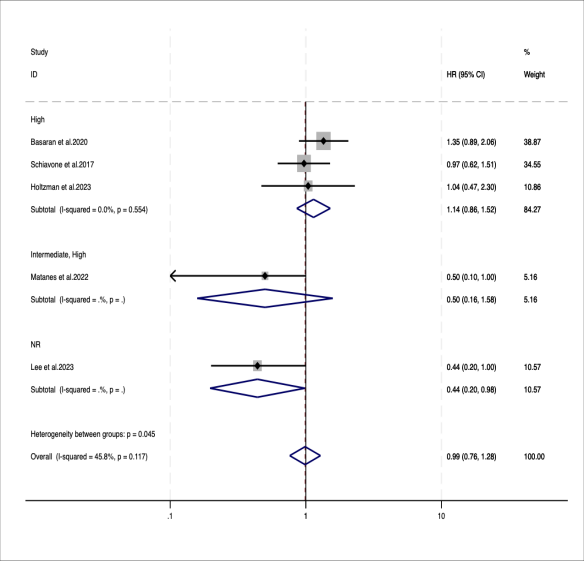

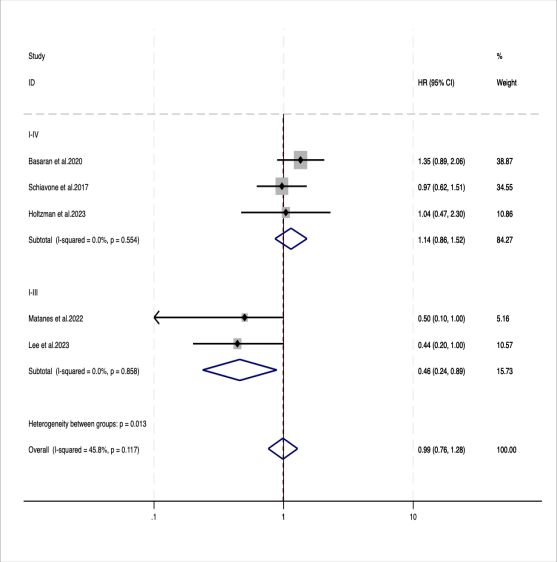


**Supplementary Figure 3. a.** Subgroup analysis of PFS by continents; **b.** Subgroup analysis of PFS by data source; **c.** Subgroup analysis of PFS by sample size; **d.** Subgroup analysis of PFS by SLNB strategy; **e.** Subgroup analysis of PFS by risk stratification; **f.** Subgroup analysis of PFS by FIGO stage
