# Supplementary material for: Long-term outcomes in patients with endometrial cancer after sentinel lymph node biopsy versus lymphadenectomy alone: a meta-analysis
Source: Front Oncol. 2025 May 20;15:1584447. doi: 10.3389/fonc.2025.1584447 (PMC12130034; doi:10.3389/fonc.2025.1584447)
Supplement: Supplementary file 2 [file Table1.docx]

**Supplementary Table 1.** Details of search strategy

**PUBMED**

| No. | Search Details | Results |
| --- | --- | --- |
| #1 | "Endometrial Neoplasms"[MeSH Terms] | 26,127 |
| #2 | "endometrium cancer"[Title/Abstract] OR "endometrial neoplasm*"[Title/Abstract] OR  "endometrial carcinoma*"[Title/Abstract] OR "endometrial cancer*"[Title/Abstract] OR  "endometrium cancer*"[Title/Abstract] OR "carcinoma of endometrium"[Title/Abstract] OR  "endometrium carcinoma*"[Title/Abstract] OR "carcinomatous endometri*"[Title/Abstract] OR  "endometrial ca"[Title/Abstract] OR "endometrial carcinogenesis"[Title/Abstract] OR  "endometrial malignanc*"[Title/Abstract] OR (("Endometrium"[MeSH Terms] OR  "Endometrium"[All Fields] OR "endometriums"[All Fields]) AND "carcinogenesis"[Title/Abstract]) | 31,305 |
| #3 | "Endometrial Neoplasms"[MeSH Terms] OR ("endometrium cancer"[Title/Abstract] OR  "endometrial neoplasm*"[Title/Abstract] OR "endometrial carcinoma*"[Title/Abstract] OR  "endometrial cancer*"[Title/Abstract] OR "endometrium cancer*"[Title/Abstract] OR  "carcinoma of endometrium"[Title/Abstract] OR "endometrium carcinoma*"[Title/Abstract] OR  "carcinomatous endometri*"[Title/Abstract] OR "endometrial ca"[Title/Abstract] OR  "endometrial carcinogenesis"[Title/Abstract] OR "endometrial malignanc*"[Title/Abstract] OR  (("Endometrium"[MeSH Terms] OR "Endometrium"[All Fields] OR "endometriums"[All Fields]) AND  "carcinogenesis"[Title/Abstract])) | 38,934 |
| #4 | "Sentinel Lymph Node Biopsy"[MeSH Terms] | 13,401 |
| #5 | "lymph node biops*"[Title/Abstract] OR "nodal biopsy"[Title/Abstract] OR  (("lymphatic vessels"[MeSH Terms] OR ("lymphatic"[All Fields] AND "vessels"[All Fields]) OR  "lymphatic vessels"[All Fields] OR "lymphatic"[All Fields] OR "lymphatics"[All Fields] OR  "lymphatic system"[MeSH Terms] OR ("lymphatic"[All Fields] AND "system"[All Fields]) OR  "lymphatic system"[All Fields]) AND "tissue biopsy"[Title/Abstract]) OR  (("lymphatic vessels"[MeSH Terms] OR ("lymphatic"[All Fields] AND "vessels"[All Fields]) OR  "lymphatic vessels"[All Fields] OR "lymphatic"[All Fields] OR "lymphatics"[All Fields] OR  "lymphatic system"[MeSH Terms] OR ("lymphatic"[All Fields] AND "system"[All Fields]) OR  "lymphatic system"[All Fields]) AND "node puncture"[Title/Abstract]) OR  "lymphatic node biopsy"[Title/Abstract] OR "lymph node puncture"[Title/Abstract] OR  "lymph node aspiration*"[Title/Abstract] OR "lymph gland biopsy"[Title/Abstract] | 11,739 |
| #6 | "Sentinel Lymph Node Biopsy"[MeSH Terms] OR ("lymph node biops*"[Title/Abstract] OR  "nodal biopsy"[Title/Abstract] OR (("lymphatic vessels"[MeSH Terms] OR  ("lymphatic"[All Fields] AND "vessels"[All Fields]) OR "lymphatic vessels"[All Fields] OR  "lymphatic"[All Fields] OR "lymphatics"[All Fields] OR "lymphatic system"[MeSH Terms] OR  ("lymphatic"[All Fields] AND "system"[All Fields]) OR "lymphatic system"[All Fields]) AND  "tissue biopsy"[Title/Abstract]) OR (("lymphatic vessels"[MeSH Terms] OR ("lymphatic"[All Fields] AND  "vessels"[All Fields]) OR "lymphatic vessels"[All Fields] OR "lymphatic"[All Fields] OR  "lymphatics"[All Fields] OR "lymphatic system"[MeSH Terms] OR ("lymphatic"[All Fields] AND  "system"[All Fields]) OR "lymphatic system"[All Fields]) AND "node puncture"[Title/Abstract]) OR  "lymphatic node biopsy"[Title/Abstract] OR "lymph node puncture"[Title/Abstract] OR  "lymph node aspiration*"[Title/Abstract] OR "lymph gland biopsy"[Title/Abstract]) | 20,242 |
| #7 | "Lymph Node Excision"[MeSH Terms] | 55,059 |
| #8 | "lymph node dissection"[Title/Abstract] OR "lymph node excisions"[Title/Abstract] OR  "lymphadenectom*"[Title/Abstract] OR "lymph node dissection*"[Title/Abstract] OR  "lymph nodal dissection"[Title/Abstract] OR "lymph node extirpation"[Title/Abstract] OR  "lymph node resection"[Title/Abstract] OR "lymphoadenectomy"[Title/Abstract] | 41,601 |
| #9 | "Lymph Node Excision"[MeSH Terms] OR "lymph node dissection"[Title/Abstract] OR  "lymph node excisions"[Title/Abstract] OR "lymphadenectom*"[Title/Abstract] OR  "lymph node dissection*"[Title/Abstract] OR "lymph nodal dissection"[Title/Abstract] OR  "lymph node extirpation"[Title/Abstract] OR "lymph node resection"[Title/Abstract] OR  "lymphoadenectomy"[Title/Abstract] | 74,580 |
| #10 | ("Endometrial Neoplasms"[MeSH Terms] OR ("endometrium cancer"[Title/Abstract] OR  "endometrial neoplasm*"[Title/Abstract] OR "endometrial carcinoma*"[Title/Abstract] OR  "endometrial cancer*"[Title/Abstract] OR "endometrium cancer*"[Title/Abstract] OR  "carcinoma of endometrium"[Title/Abstract] OR "endometrium carcinoma*"[Title/Abstract] OR  "carcinomatous endometri*"[Title/Abstract] OR "endometrial ca"[Title/Abstract] OR  "endometrial carcinogenesis"[Title/Abstract] OR "endometrial malignanc*"[Title/Abstract] OR  (("Endometrium"[MeSH Terms] OR "Endometrium"[All Fields] OR "endometriums"[All Fields]) AND  "carcinogenesis"[Title/Abstract]))) AND ("Sentinel Lymph Node Biopsy"[MeSH Terms] OR  ("lymph node biops*"[Title/Abstract] OR "nodal biopsy"[Title/Abstract] OR (("lymphatic vessels"[MeSH Terms] OR  ("lymphatic"[All Fields] AND "vessels"[All Fields]) OR "lymphatic vessels"[All Fields] OR "lymphatic"[All Fields] OR  "lymphatics"[All Fields] OR "lymphatic system"[MeSH Terms] OR ("lymphatic"[All Fields] AND "system"[All Fields]) OR  "lymphatic system"[All Fields]) AND "tissue biopsy"[Title/Abstract]) OR (("lymphatic vessels"[MeSH Terms] OR  ("lymphatic"[All Fields] AND "vessels"[All Fields]) OR "lymphatic vessels"[All Fields] OR "lymphatic"[All Fields] OR  "lymphatics"[All Fields] OR "lymphatic system"[MeSH Terms] OR ("lymphatic"[All Fields] AND "system"[All Fields]) OR  "lymphatic system"[All Fields]) AND "node puncture"[Title/Abstract]) OR "lymphatic node biopsy"[Title/Abstract] OR  "lymph node puncture"[Title/Abstract] OR "lymph node aspiration*"[Title/Abstract] OR "lymph gland biopsy"[Title/Abstract])) AND  ("Lymph Node Excision"[MeSH Terms] OR ("lymph node dissection"[Title/Abstract] OR "lymph node excisions"[Title/Abstract] OR  "lymphadenectom*"[Title/Abstract] OR "lymph node dissection*"[Title/Abstract] OR "lymph nodal dissection"[Title/Abstract] OR  "lymph node extirpation"[Title/Abstract] OR "lymph node resection"[Title/Abstract] OR "lymphoadenectomy"[Title/Abstract])) | 587 |

**EMBASE**

| No. | Query | Results |
| --- | --- | --- |
| #1 | 'lymph node dissection'/exp | 101080 |
| #2 | 'lymph node excision*':ab,ti OR lymphadenectom*:ab,ti OR 'lymph node dissection*':ab,ti OR 'lymph nodal dissection':ab,ti OR 'lymph node extirpation':ab,ti OR 'lymph node resection':ab,ti OR lymphoadenectomy:ab,ti | 64745 |
| #3 | #1 OR #2 | 111716 |
| #4 | 'lymph node biopsy'/exp | 41590 |
| #5 | 'sentinel lymph node biopsy':ab,ti OR 'lymph gland biopsy':ab,ti OR 'lymph node aspiration*':ab,ti OR 'lymph node puncture':ab,ti OR 'lymphatic node biopsy':ab,ti OR 'lymphatic node puncture':ab,ti OR 'lymphatic tissue biopsy':ab,ti OR 'nodal biopsy':ab,ti OR 'lymph node biops*':ab,ti | 18578 |
| #6 | #4 OR #5 | 44690 |
| #7 | 'endometrium cancer'/exp | 66426 |
| #8 | 'endometrium tumor':ab,ti OR 'endometrial carcinoma*':ab,ti OR 'endometrial cancer*':ab,ti OR 'endometrium cancer*':ab,ti OR 'carcinoma of endometrium':ab,ti OR 'endometrium carcinoma*':ab,ti OR 'carcinomatous endometri*':ab,ti OR 'endometrial ca':ab,ti OR 'endometrial carcinogenesis':ab,ti OR 'endometrial malignanc*':ab,ti OR 'endometrium carcinogenesis':ab,ti | 46174 |
| #9 | #7 OR #8 | 73710 |
| #10 | #3 AND #6 AND #9 | 678 |

**COCHRANE**

| No. | Search Hits Hit Hits | Results |
| --- | --- | --- |
| #1 | MeSH descriptor: [Endometrial Neoplasms] explode all trees | 1027 |
| #2 | (Endometrium Cancer or Endometrial Neoplasm* or Endometrial Carcinoma* or Endometrial Cancer* or Endometrium Cancer* or Carcinoma of Endometrium or Endometrium Carcinoma*):ti,ab,kw | 3280 |
| #3 | #1 or #2 | 3334 |
| #4 | MeSH descriptor: [Sentinel Lymph Node Biopsy] explode all trees | 514 |
| #5 | (lymph gland biopsy or lymph node aspiration* or lymph node puncture or lymphatic node biopsy or lymphatic node puncture or lymphatic tissue biopsy or nodal biopsy or lymph node biops*):ti,ab,kw | 2628 |
| #6 | #4 or #5 | 2628 |
| #7 | MeSH descriptor: [Lymph Node Excision] explode all trees | 2045 |
| #8 | (Lymph Node Excisions or Lymphadenectom* or Lymph Node Dissection* or lymph nodal dissection or lymph node extirpation or lymph node resection or lymphoadenectomy):ti,ab,kw | 6407 |
| #9 | #7 or #8 | 7013 |
| #10 | #3 and #6 and #9 | 54 |

**WEB OF SCIENCE**

| No. | Query | Results |
| --- | --- | --- |
| #1 | Endometrium Cancer (Topic) or Endometrial Neoplasm* (Topic) or Endometrial  Carcinoma* (Topic) or Endometrial Cancer* (Topic) or Endometrium Cancer* (Topic) or Carcinoma of Endometrium (Topic) or Endometrium Carcinoma* (Topic) or carcinomatous endometri* (Topic) or endometrial Ca (Topic) or endometrial carcinogenesis (Topic) or endometrial malignanc* (Topic) or endometrium | 97889 |
| #2 | Sentinel Lymph Node Biopsy (Topic) or lymph gland biopsy (Topic) or lymph node aspiration* (Topic) or lymph node puncture (Topic) or lymphatic node biopsy (Topic) or lymphatic node puncture (Topic) or lymphatic tissue biopsy (Topic) or nodal biopsy (Topic) or lymph node biops* (Topic) and Preprint Citation Index (Exclude - Database) | 166,895 |
| #3 | Lymph Node Excision* (Topic) or lymph node dissection (Topic) or Lymphadenectom* (Topic) or Lymph Node Dissection* (Topic) or lymph nodal dissection (Topic) or lymph node extirpation (Topic) or lymph node resection (Topic) or lymphoadenectomy (Topic) and Preprint Citation Index (Exclude - Database) | 130,952 |
| #4 | #1 AND #2 AND #3 and Preprint Citation Index (Exclude - Database) | 1101 |
