# Supplementary material for: Long-term outcomes in patients with endometrial cancer after sentinel lymph node biopsy versus lymphadenectomy alone: a meta-analysis
Source: Front Oncol. 2025 May 20;15:1584447. doi: 10.3389/fonc.2025.1584447 (PMC12130034; doi:10.3389/fonc.2025.1584447)
Supplement: Supplementary file 3 [file Table2.docx]

**Supplementary Table 2.** Characteristics of the 13 studies

| **Author,Year** | **Country** | **Continent** | **Study design** | **Institution** | **Study period** | **SLN** | **LND** | **Age(SLN vs LND)** |
| --- | --- | --- | --- | --- | --- | --- | --- | --- |
| Basaran et al.2020 | USA | North America | Retrospective cohort study | Memorial Sloan Kettering Cancer Center | 01/01/1996-12/31/2017 | 79 | 166 | 66 (51-89)vs68(44-87) |
| Bogani et al.2020 | Italy | Europe | Retrospective cohort study | Fondazione IRCCS Istituto dei Tumori, Milano and University of Insubria – Del Ponte Hospital, Varese and University of Genoa | 01/01/2006-12/31/2016 | 90 | 180 | 60.88 (11.0)vs62.5 (8.8) |
| Nasioudis et al.2021 | USA | North America | Retrospective cohort study | National Cancer Database | 01/2012-12/2015 | 109 | 1323 | NR |
| Matanes et al.2022 | Canada | North America | Retrospective cohort study | Segal Cancer Center of the Jewish General Hospital, Montreal, Canada | 12/r 2007-08/2017 | 74 | 84 | 71 (65–87)vs75 (65–91) |
| Lee et al.2023 | Korea | Asia | Retrospective cohort study | Yonsei Cancer Center, Seoul, Republic of Korea | 2006-2013,2014-2021 | 257 | 645 | 53.5±1vs54.1±10 |
| Nahshon et al.2023 | Israel | Asia | Retrospective cohort study | NR | 01/01/2010-12/31/2019 | 6019 | 6019 | 62.2±10.7vs62.1±10.5 |
| Schiavone et al.2017 | USA | North America | Retrospective cohort study | Memorial Sloan Kettering Cancer Center | 01/ 2005-07/2015 | 153 | 95 | 65 (45-89)vs68 (45-85) |
| Nasioudis et al.2020 | USA | North America | Retrospective cohort study | National Cancer Data Base | 2012-2015 | 460 | 920 | NR |
| Nasioudis et al.2021 | USA | North America | Retrospective cohort study | National Cancer Database | 2012-2015 | 3149 | 9861 | NR |
| Brezinov et al.2022 | Israel | Asia | Retrospective cohort study | Kaplan medicalcenter,Israeli Gynecologic Oncology Group | 2013-2018,2002-2014 | 138 | 1022 | 66.4(±9.7)vs65.1(±10.2) |
| Matsuo et al.2022 | USA | North America | Retrospective cohort study | National Cancer Institute’s Surveil- lance, Epidemiology, and End Results (SEER) Program | 2010-2018 | 340 | 4915 | 64 (56-70)vs64 (57-71) |
| Ting et al.2022 | China | Asia | Retrospective cohort study | NR | 01/2008-05/2021 | 62 | 272 | 57.3±10.6vs55.5±10.0 |
| Holtzman et al.2023 | USA | North America | Retrospective cohort study | Icahn School of Medicine at Mount Sinai | 01/01/2014-09/01/2020 | 46 | 143 | 63.5 (50, 92)vs66 (38, 90) |

Continue table

| **Author,Year** | **BMI(SLN vs LND)** | **FIGO stage** | **Grade** | **Histology** | **Risk stratification** | **Approach** | **SLN procedure** | **Data sources** |
| --- | --- | --- | --- | --- | --- | --- | --- | --- |
| Basaran et al.2020 | 30.6(18.2-53.4)vs29.2(17.2-60.3) | I-IV | NR | Serous Uterine Carcinoma | NR | Laparotomy,minimally invasive surgery | Cervical injection of blue dye or indocyanine green | curves |
| Bogani et al.2020 | 26.36 (4.1)vs27.3 (6.7) | I-III | G1-3 | Endometrioid,Non-endometrioid | Low,Intermediate, High | Laparoscopy,Open surgery | NR | curves |
| Nasioudis et al.2021 | NR | IIIC | NR | Endometrioid,Serous,clear cell,carcinosarcoma ,Mixed/other | NR | Laparoscopic,Robotic assisted | NR | Multivariate analysis |
| Matanes et al.2022 | 29 (18–51)vs28 (18–63) | I-III | G1-3 | Endometrioid,Serous,Clear cell,Carcinosarcoma | Intermediate, High | Robotically assisted | NR | Multivariate analysis |
| Lee et al.2023 | 25.2±4vs25.2±4 | I-III | G1-3 | Endometrioid,Serous,Carcinosarcoma,other | NR | laparoscopic,Robotically assisted | Cervical injection of indocyanine green | Multivariate analysis |
| Nahshon et al.2023 | NR | I-IV | G1-3 | Endometrail,Non-endometrial | NR | NR | NR | Multivariate analysis |
| Schiavone et al.2017 | 29.6 (17.6–53.4)vs29.3 (17.2–60.3) | I-IV | NR | Serous Uterine Carcinoma | NR | laparotomy or laparoscopy, with or without the use of the robotic | Cervical injection of blue dye or indocyanine green | univariate analysis |
| Nasioudis et al.2020 | NR | I-II | NR | Grade 3 endometrioid,serous,clear cell,carcinosarcoma | High | Laparoscopic,Robotic-assisted | Cervical injection of indocyanine green | Multivariate analysis |
| Nasioudis et al.2021 | NR | IA,IB,INOS | G1-3,Unknown | Endometrioid | NR | Laparoscopic,Robotic-assisted | NR | Multivariate analysis |
| Brezinov et al.2022 | NR | I-III | G1-3 | Endometrioid,Serous papillary,Clear cell,Carcinosarcoma | Low,high | laparotomy,laparoscopy | Cervical injection of blue dye and Tc99 | curves |
| Matsuo et al.2022 | NR | II | NR | Endometrioid,Serous,Clear cell,Carcinosarcoma,Mixed,Others | NR | NR | NR | Multivariate analysis |
| Ting et al.2022 | 28.1±4.4vs27.0±6.1 | I-IV | G1-3 | NR | NR | laparotomic, laparoscopic, robotic approach. | Cervical injection of indocyanine green | curves |
| Holtzman et al.2023 | 30.6 (19.1, 56.1)vs29.3 (18.1, 51.0) | I-IV | NR | Endometroid,Clear cell,Serous,Carcinosarcoma,Other | High | Open,Robotic-Assisted,Laparoscopic | NR | Multivariate analysis |
