# Supplementary material for: Long-term outcomes in patients with endometrial cancer after sentinel lymph node biopsy versus lymphadenectomy alone: a meta-analysis
Source: Front Oncol. 2025 May 20;15:1584447. doi: 10.3389/fonc.2025.1584447 (PMC12130034; doi:10.3389/fonc.2025.1584447)
Supplement: Supplementary file 4 [file Table3.docx]

**Supplementary Table 3.** Descriptive characteristics of the assessment

| **Study** | **Selection** | | | | **Comparability** | **Outcome** | | | **Quality scores** |
| --- | --- | --- | --- | --- | --- | --- | --- | --- | --- |
|  | **Representativeness of the exposed cohort** | **Selection of the nonexposed cohort** | **Ascertainment of exposure** | **Demonstration that outcome of interest was not present at start of study** | **Comparability of cohorts on the basis of the design or analysis** | **Assessment of outcome** | **Was follow-up long enough for outcomes to occur** | **Adequacy of follow up of cohorts** |  |
| Basaran et al.2020 | **1** | **1** | **1** | **1** | **1** | **1** | **1** | **1** | 8 |
| Bogani et al.2020 | **1** | **1** | **-** | **1** | **1** | **1** | **1** | **1** | 7 |
| Nasioudis et al.2021 | **1** | **1** | **-** | **1** | **2** | **1** | **1** | **1** | 8 |
| Matanes et al.2022 | **1** | **1** | **-** | **1** | **2** | **1** | **1** | **1** | 8 |
| Lee et al.2023 | **1** | **1** | **1** | **1** | **2** | **1** | **1** | **1** | 9 |
| Nahshon et al.2023 | **1** | **1** | **-** | **1** | **2** | **1** | **1** | **1** | 8 |
| Schiavone et al.2017 | **1** | **1** | **1** | **1** | **1** | **1** | **1** | **1** | 8 |
| Nasioudis et al.2020 | **1** | **1** | **1** | **1** | **2** | **1** | **1** | **1** | 9 |
| Nasioudis et al.2021 | **1** | **1** | **-** | **1** | **2** | **1** | **1** | **1** | 8 |
| Brezinov et al.2022 | **1** | **-** | **1** | **1** | **1** | **1** | **1** | **1** | 7 |
| Matsuo et al.2022 | **1** | **1** | **-** | **1** | **2** | **1** | **1** | **1** | 8 |
| Ting et al.2022 | **1** | **1** | **1** | **1** | **1** | **1** | **1** | **1** | 8 |
| Holtzman et al.2023 | **1** | **1** | **-** | **1** | **2** | **1** | **1** | **1** | 8 |
